# Describing Patterns of Known Domestic Abuse Among Different Ethnic Groups

**DOI:** 10.3389/fpsyg.2022.917543

**Published:** 2022-06-01

**Authors:** Matthew Bland, Ruth Weir, Olumide Adisa, Katherine Allen, Joana Ferreira, Dev Rup Maitra

**Affiliations:** ^1^Institute of Criminology, University of Cambridge, Cambridge, United Kingdom; ^2^Centre for Abuse Research, University of Suffolk, Ipswich, United Kingdom; ^3^Violence and Society Centre, City, University of London, London, United Kingdom

**Keywords:** domestic abuse, domestic violence, intimate partner abuse, disproportionality, crime harm, racially minoritized communities

## Abstract

Domestic abuse perpetration remains a major threat to public health, safety and wellbeing, causing serious harms and contributing significantly to overall crime globally. In the United Kingdom, research links the crime type to high economic and social costs. In the last 10 years, our collective knowledge of domestic abuse has grown in conjunction with its prioritisation in government policy. Several innovative studies have built a picture of the most serious cases and overall patterns of abuse but to date, examination of these trends by ethnic groups has been limited despite increasing attention to disproportionality in racially minoritised communities in criminal justice system outcomes. In this article we aimed to address this issue through the analysis of 150,000 domestic abuse records kept by police forces in England. Using descriptive statistics, we examined the relative distributions of different ethnicities by suspected offending rate, investigative outcome and crime harm. We found two patterns of note: firstly, that suspects from several categories of minoritized communities are consistently over-represented compared to the White British population among most harmful cases, and secondly, that in Asian communities, offences are less frequently “solved.” We discuss the implications for future research and practice.

## Introduction

Domestic abuse perpetration is a major threat to public health, safety and wellbeing in the 21st century. It causes serious harms and contributes significantly to overall crime. The recently legislated definition of this crime includes physical, sexual, violent, controlling, economic and psychological forms of abuse (UK Government, 2021) Office for National Statistics (ONS) data for the year ending March 2020 states that an estimated 5.5% of adults aged 16–74 were subjected to domestic abuse in the previous 12 months, and 357 domestic homicides were recorded by police between March 2017 and March 2019 (Office for National Statistics [ONS], 2020). Meanwhile, over one-third (35%) of all violence against the person offences, and around 16% of sexual offences–recorded by England and Wales police in the year ending March 2020–were flagged as domestic abuse related (*ibid*).

Domestic abuse has emerged as a significant policing priority over the past decade, particularly following scrutiny by the national police oversight body (Her Majesty’s Inspectorate of Constabulary, Fire and Rescue Services [HMICFRS], 2014) regarding failings in the police response to victims. However, given reduced police capacity in the wake of significant budget cuts, and rising demand for interventions, police forces are under pressure to ensure that finite resources are being directed in the most effective and targetted way possible.

Recent research on the estimated economic and social costs of domestic abuse found that, for the year ending 31 March 2017, the overall cost of domestic abuse amounted to £66 billion (Oliver et al., 2019). This sum includes an estimated £47 billion associated with the considerable emotional and physical harms sustained by victims, as well as costs to the economy linked to reduced economic productivity and output (£14 billion), and costs for health service (£2.3 billion) and police (£1.3 billion) (Oliver et al., 2019). The magnitude of individual, social and economic harms incurred because of domestic abuse underlines the need to tackle the root of the problem, identifying and working with those perpetrators likely to cause the most harm.

When responding to domestic abuse among racially minoritised communities, it is crucial to account for historical and social context, and how this may affect confidence and trust in the police and criminal justice system and willingness to report domestic abuse. Black, Asian and other racially minoritised people continue to be over-represented in the criminal justice system in England and Wales, and to experience disparate outcomes. For example, the Lammy (2017) Review found that, while making up only 14% of the population, Black, Asian and other racially minoritised individuals made up 25% of prisoners, and more than 40% of young people in custody. This disproportionality extends to pronounced differences in sentencing for some crimes; for example, for drugs offences, other racially minoritised individuals were 240% more likely to receive a prison sentence than White offenders (Lammy, 2017).

Concerningly, the Crown Prosecution Service has also identified significant discrepancies in the prosecution and conviction rates for domestic abuse, with a higher prosecution rate for Black, Chinese and “Other” defendants (Lammy, 2017). This disparity indicates that other racially minoritised defendants are disproportionately likely to face imprisonment, and perhaps accordingly may be less likely to have the opportunity to access evidence-based and rehabilitative community interventions such as Respect-accredited perpetrator programmes.

These differences in treatment throughout the criminal justice system impact not only offenders but all those racially minoritised individuals disproportionately affected by policing practices such as Stop and Search, or through the increased arrest rate for Black and Mixed ethnic background people (Lammy, 2017). The pervasive “racialisation” of crime by the media, and the perception that criminal justice system structures and procedures selectively “target and criminalise” Black, Asian and other racially minoritised people (Fekete, 2018, p. 77), could diminish confidence in police among racially minoritised people experiencing domestic abuse, and may make it less likely that these victims will trust police to intervene and deliver just outcomes (see Adisa and Allen, 2020).

The racial disparities which exist at each stage of the criminal justice system have implications not only for the offender but for the wider community, resulting in a “trust deficit” that reduces police’s ability to safeguard survivors, disrupt perpetration and hold those using harmful behaviours to account (Lammy, 2017: 29).

When designing, commissioning and evaluating interventions for racially minoritised individuals using harmful behaviours, the legacies of this ingrained inequity must be considered. For instance, professionals from Black, Asian and other racially minoritised communities note that the use of the term “perpetrator” may be experienced as alienating and associated with racialised stereotypes about criminality, deterring people from seeking help to change their harmful behaviours (see Govier and Verwoerd, 2004 for a discussion on aspects of how labelling language can cause false dichotomies). Additionally, culturally specific interventions are lacking in the current landscape of perpetrator interventions which limit our understanding of “What works” and “for whom” within Black, Asian and racially minoritised communities (Adisa and Allen, 2020).

Currently, tailored provision for Black, Asian and other racially minoritised people seeking to end their use of harmful behaviours remains sparse; a recent rapid review of non-mandated interventions for those using abusive behaviours in intimate relationships did not include any culturally specific or specialised programmes for Black, Asian or racially minoritised people (Callaghan et al., 2020).

### The Current Study

This research aims to use domestic abuse crime data to assess the relationships and patterns between levels of harm and potential predictor variables. This is a descriptive study which aims to set a foundation for future analysis. Its findings may aid future policy decisions before they influence future research, including the refinement of prevention and risk assessment procedures.

This research is exploratory in nature, dealing with a cross-sectional dataset. As this study was part of a broader framework of research, our research questions did not venture beyond the descriptive–seeking to establish a “baseline” profile of the issue of harm and its distribution across ethnicities. It sought to address the following questions in particular:

• RQ1–What is the profile of domestic abuse suspects by ethnicity?

• RQ2–What is the profile of crime harm, overall and by ethnicity?

• RQ3–What is the profile of risk assessment by ethnicity?

• RQ4–What is the profile of investigative outcome by ethnicity?

• RQ5–What is the contribution of different ethnicities to the “power few” most harmful suspects?

## Materials and Methods

### Participants

Datasets were supplied from three English police forces, anonymised in this article as Forces A, B, and C. A data specification and follow up meeting was provided to each force through the data collection process, ensuring some consistency of format in the datasets received. Although data recording for crimes–and domestic abuse in particular–is subject to national guidelines, at an individual record level there are numerous discrepancies to manage when aggregating data of this nature such as differences in code lists.

### Materials

The consistent variables we were able to secure comprised of: (1) Offence ID number, (2) earliest date upon which the crime took place, (3) Home Office Counting Rule code^1^, (4) Crime Classification description, (5) Investigation outcome, (6) Suspect ID number (where applicable), (7) suspect age, (8) suspect ethnicity as defined by themselves (known as self-defined ethnicity), (9) suspect ethnicity as defined by the recording officer, (10) suspect sex and various indicators of suspect’s prior criminal history for domestic and non-domestic crimes.

### Procedure

Each participating police force was supplied with a data template which was explained at virtual meetings with the research team. Each dataset was supplied in Microsoft Excel format and subsequently synthesised into an amalgamated dataset of consistent variables. One calculated variable was added to the dataset for the measurement of harm. This variable took the Home Office Crime Recording classification as its source and used the Cambridge Crime Harm Index (CCHI) (Sherman et al., 2016) as its reference. The CCHI weights crime classifications by days, with days relating to sentencing guidelines. Each weight refers to the minimum sentence a court may issue: for example, a robbery has a minimum sentence of 1 year in prison so the weight is 365 (days). As its authors have argued (and is subsequently discussed in Sherman (2020), the CCHI offers a way of comparing crime patterns taking into account that each crime is different. The harm captured by CCHI is against the state and is formulated in a consistent and democratic framework. The CCHI has been used by multiple published studies of domestic abuse [see (Bland and Ariel, 2015); 2020 for examples]. Bland and Ariel (2020) sets out the case for CCHI being current “superior” method for measuring harm for English or Welsh crime datasets.

### Data Analysis

The majority of analysis were undertaken in Microsoft Excel 2019 using pivot tables to generate descriptive statistics. We use *z*-tests to compare the proportional distribution of ethnicities, which were calculated with the online calculator available from Social Science Statistics^2^, which includes z-statistics and accompanying *p*-values.

## Results

### RQ1–What Is the Proportion of Domestic Abuse Crimes by Ethnicity?

A moderate proportion of self-defined ethnicity data are unrecorded, either due to the suspect being unidentified, refusing to answer the question or the police failing to record the answer. Force A reported 28% of cases with no self-defined ethnicity by the suspect. In Force B, this proportion was 10% and in Force C it was 51%.

For the purposes of this profiling, these records were excluded but clearly the true answers may skew our findings, even in the most optimistic case. We are unable to decipher if the gaps in recording are systematic or random and so we urge a note of caution in the interpretation of these findings.

**FIGURE 1:**
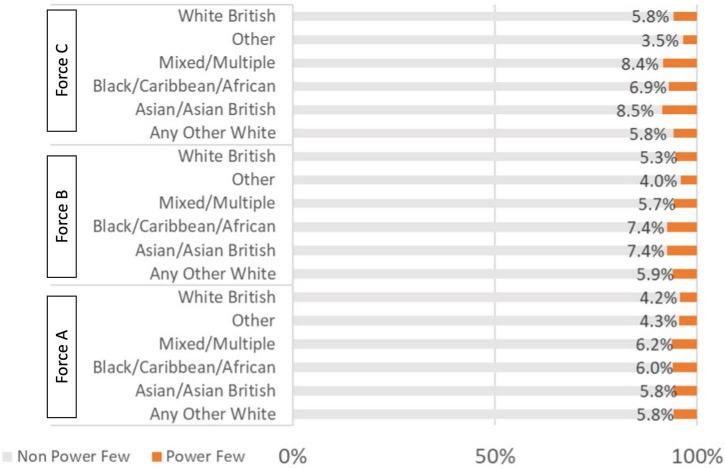
Comparison of repeat suspect rates across ethnicity bands.

Figure 1 shows that there is no distinct or obvious pattern of higher repeat offending rates in Black/Caribbean/African suspects compared with White British suspects. These analyses are based on our overall crime dataset, so repeat offenders of the same recorded ethnicity may skew results. We explored the extent of repeat offending on the offender subset, therefore controlling for high volumes of repeat offenders.

### RQ2–What Is the Profile of Crime Harm, Overall and by Ethnicity?

**FIGURE 2:**
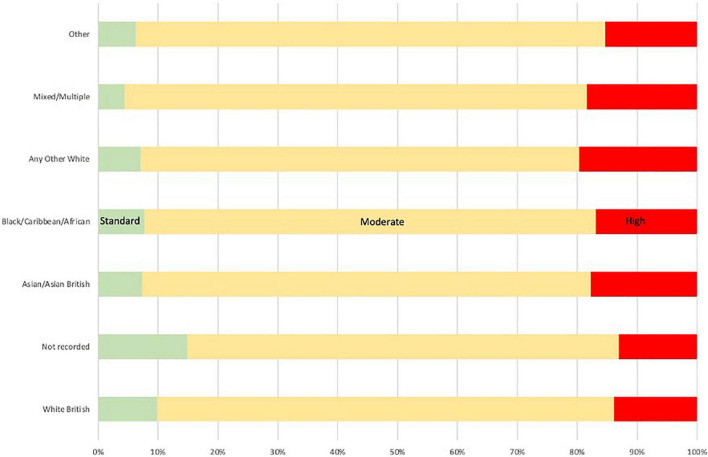
Distribution of CCHI totals among suspects.

Typically, analyses that utilise the CCHI are not normally distributed (see Bland and Ariel, 2015, 2020; Barnham et al., 2017; Kerr et al., 2017). This is also the case with our dataset, which represents something approximating a Pareto distribution^3^. As Figure 2 shows, most suspects across the three datasets accumulated CCHI totals equivalent to less than six months in prison. There is not a universal Pareto distribution however–note the peak around 1825 days (5 years) which is linked to the minimum sentence for grievous bodily harm offences.

This distribution mirrors that seen in previous studies in this area (see Bland and Ariel, 2015, 2020; Barnham et al., 2017). In simplistic terms, a small proportion of suspects are associated with a greater proportion of harm. In these three datasets combined this trend is that 5% of suspects account for 65% overall harm. This issue is explored in more detail in RQ5.

The measure of central tendency in the data is affected by this distribution, which includes some extreme outliers. The mean number of CCHI days is 177 (*SD* = 538). The median is a more accurate reflection of the centre of the dataset at 10 days. The central point holds true across different ethnicity bandings within the force jurisdictions The exception was notably Force A, where median CCHI totals were approximately half of those in Force B and Force C for every ethnicity banding. In practical terms, we might infer that the “typical” cumulative harm of domestic abuse offenders does not rise above the level of an actual bodily harm–a violent crime which is to the detriment of the victim without causing serious physical injury by itself.

### RQ3–What Is the Profile of Risk Assessment by Ethnicity?

**FIGURE 3:**
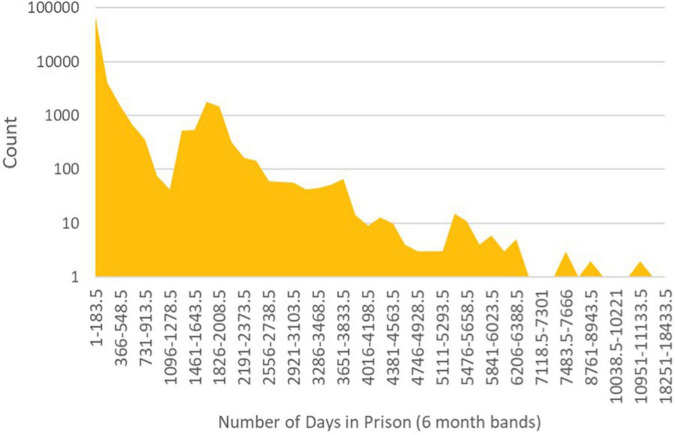
Proportions of risk assessment score by ethnicity banding.

Force A was the only force to supply us with risk assessment gradings. In total, 74% of these were at the “moderate” risk level, 15% at the “high” risk level and the remaining 10% at “standard” risk. Almost a third of these records had no recorded suspect ethnicity, so we emphasise caution in the findings and note that among these “blank ethnicity” cases, 15% were “standard” risk. Figure 3 shows the full breakdown.

It is difficult to draw meaningful conclusions from these data based on the descriptive analysis alone. They relate to just one force, and the smallest dataset among those we received. They do not indicate any stark disproportionate differences in gradings between differing ethnicity groups but there are differences. 13.8% of white British cases are identified as “high risk.” Proportionally, all other bandings have higher rates of “high risk” grading, with “any other white” the most different at almost 1.5 times the rate.

### RQ4–What Is the Profile of Investigative Outcome by Ethnicity?

All domestic abuse crimes reported to the police are investigated and assigned an “outcome code” based on the results of that investigation. Broadly, the 22 available codes are divided into two categories which might be described as “solved” and “unsolved.” Solved cases include charging the suspect to court, issuing a caution or community resolution. Unsolved codes are divided into differing reasons for that outcome, such as a different organisation being passed the case or the victim being unwilling to support a prosecution. Police forces are commonly assessed on their “solved rates,” the proportion of crimes which they obtain a positive outcome for, as an indicator of their performance.

In Force A, the “solved rate” for cases involving “White British” suspects was 13.4%, compared to 16.4% in Force B and 18.9% in Force C. There was little variation between these rates and those of minoritized communities. “Black/Caribbean/African” suspect cases were “solved” in 13.3, 16.2, and 18% of the time in Forces A–C, respectively. One pattern that was repeated was that cases involving “Asian/Asian British” suspects are solved at between 0.79 and 0.86 times the rate of “White British” cases. Indeed, the solved rate for cases with “Asian/Asian British” suspects is nearly always lower than all other bandings.

### RQ5–What Is the Contribution of Different Ethnicities to the “Power Few” Most Harmful Suspects?

In RQ2, we identified that the distribution of harm in our datasets broadly mirrors a Pareto distribution mirroring previous work on domestic abuse harm. Specifically, we highlighted that 5% of suspects correlate with 65% of harm. This is consistent with the concept of “the power few” (Sherman, 2007)–the few offenders who offer the most powerful opportunities for harm reduction.

When dividing the aggregated data into three datasets, the “power” of the power few in each force is slightly different. In Force A, 544 suspects equate to the top 5% most harmful suspects. Together, these 544 represent 78% of cumulative harm in Force A. In Force B, the 5% most harmful suspects is made up of 1,413 individuals who collectively account for 54% of total CCHI days. In Force C, the total is 2,601 suspects who represent 59% of harm.

In Force B and Force C, a total score of 1,825 CCHI days (equivalent to a grievous bodily harm offence) would mean a suspect is included in the “power few.” In Force A, the distribution of harm is more acute. A score of 400 days or above would place a suspect in the top 5%. Nevertheless, we have treated each force as distinct to reflect the patterns within each jurisdiction’s most harmful suspects.

**FIGURE 4:**
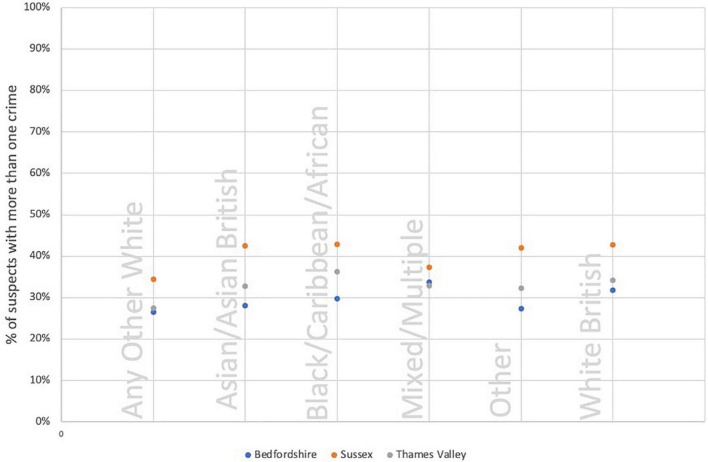
Proportion of suspects within the power few, by ethnicity banding.

These analyses show that “Asian/Asian British,” “Black/Caribbean/African” and “Mixed/Multiple” bandings are consistently over-represented in the most harmful group of suspects than we might expect if all things were equal. The baseline distribution of the “power few” is that just 5% of suspects are within this category. So, our starting hypothesis is that each ethnicity banding will reflect this equally. Figure 4 shows the proportion of each ethnicity banding that are within the “power few.”

Proportions as small as these can be difficult to interpret visually. We might notice that “Asian/Asian British” proportions are higher in two forces but how much stock to place in this difference is harder to determine without inferential statistics. We undertook *z*-tests for two population proportions to test the hypothesis that these proportions were different from each other in a generalisable sense. From these we may conclude that we may accept that there are real differences between the proportions of “White British” and all three of “Asian/Asian British,” “Black/Caribbean/African” and “Mixed/Multiple” suspects in the power few. These differences are universally consistent with a higher proportion of suspects in the latter three categories.

## Discussion

This study is the latest in a series of analyses of domestic abuse crime data (see Bland and Ariel, 2015, 2020; Barnham et al., 2017; Kerr et al., 2017; Turner et al., 2019; Weir, 2019) but the first to explore the distribution of harm among ethnicities. Like all crime data studies, the data source is flawed. Domestic abuse is underreported in crime data (Office for National Statistics [ONS], 2019) and it if patterns in the United Kingdom replicate those found in the United States (Holliday et al., 2020) then it is likely that the underreporting is even more pronounced in ethnicity-based analyses like this one. To compound this issue, our research suggests a problem with the recording of self-defined ethnicity in police data. For half the domestic abuse suspect records in a large police organisation to be missing any self-defined ethnicity information is a substantial gap that policymakers need to attend to.

Notwithstanding these issues, our study presents the scholarly attempt to dissect patterns by ethnicity in reported domestic abuse. It shows general homogeneity in trends across ethnicities in respect of repeat suspect rates, median harm and risk assessments. Two differences in ethnicity profile pique our interest. The first is that “Asian/Asian British” suspects are consistently among the lowest for proportion of cases solved. We must be cautious–we have only analysed three of the 43 forces in England and Wales, but it is notable that this ethnicity is in the lowest two in each of the three we have examined. The second is that “Asian/Asian British,” “Black/Caribbean/African” and “Mixed/Multiple” ethnicities all feature more frequently in the “power few” group of most harmful offenders more than “White British” suspects. “White British” suspects make up the largest proportion of victims and suspects of domestic abuse by virtue of the fact that this category is the largest ethnicity classification in the United Kingdom (80.5% of the United Kingdom population recorded in the 2011 census was White British) and the results of the Crime Survey of England and Wales (Office for National Statistics [ONS], 2021). The ONS survey reports on victims, not suspects so we cannot draw precise comparisons here, as our research discusses suspects. But the fact that we have identified statistically significant differences in the expected proportions of suspects in “Asian/Asian British” and “Black/Caribbean/African” compared to “White British” in each of the three police jurisdictions we studied is notable and worthy of further exploration. If domestic abuse dyads are predominantly ethnically homogenous (and this has not been rigorously established), and if underreporting is greater in minority ethnic communities, then why are minority ethnic suspects more frequently making up the most harmful cohort of offenders? One explanation may be that these cohorts are, by definition, more visible to police. Serious physical crimes (such as grievous bodily harm and homicide), leave more evident traces and so underreporting becomes less of a factor. But this is merely a hypothesis.

Our findings are based on data with limitations. Police administrative records do not represent all domestic abuse that happens in society. The differing rates of ethnicity recording is also problematic for the drawing of robust inferences but we can still state that more research is urgently needed to investigate the disparity in ethnicity composition of the most harmful suspects. The same research should be undertaken for victims of domestic abuse. All 43 police agencies in England and Wales record information on these individuals, so such research is far from infeasible. Indeed, it is essential if we are to confront important questions for policymaking. A more widespread analytic review of data gaps is the first logical step to developing our understanding, but it is also likely that researchers will need to consult wider datasets than just police recorded crime. Community surveys such as the Crime Survey of England and Wales (see Ariel and Bland, 2019 for a comparison of this measure and police records).

When it comes to domestic abuse responses, it appears that ethnicity is an important variable. Simply adopting a “colour blind” or “one size fits all” approach means that racially minoritised people’s specific needs and sensitivities too often go unrecognised and unfulfilled. To ensure equal protection from harm, and equal access to justice, it is incumbent on those designing, commissioning and evaluating programmes to explicitly consider the needs of different groups and make sure that these are embedded at each stage of programme development.

## Data Availability Statement

The data analysed in this study is subject to the following licences/restrictions: Data are subject to General Data Protection Regulation provisions as outlined in individual Information Sharing Agreements with the data providers. Requests to access these datasets should be directed to OA, o.adisa@uos.ac.uk.

## Ethics Statement

The research was conducted having been augmented by the University of Suffolk’s Research Ethics Committee. Research undertaken at the University of Suffolk complies with the UK Research Integrity Office (UKRIO) Code of Practice for Research (2021).

## Author Contributions

MB conducted all data analyses and was the architect of this manuscript. MB, RW, and OA collaborated on the design of this research following an original idea by OA. OA secured the funding and was responsible for overall administration of the project until her maternity leave (congratulations!) when DM took over. KA and JF undertook research of literature and contributed to the drafting of this manuscript. All authors contributed to the article and approved the submitted version.

## Conflict of Interest

The authors declare that the research was conducted in the absence of any commercial or financial relationships that could be construed as a potential conflict of interest.

## Publisher’s Note

All claims expressed in this article are solely those of the authors and do not necessarily represent those of their affiliated organizations, or those of the publisher, the editors and the reviewers. Any product that may be evaluated in this article, or claim that may be made by its manufacturer, is not guaranteed or endorsed by the publisher.

## Footnotes

1 The UK Home Office regulates the crimes recorded by police forces under one catalog of codes and definitions, known as the Home Office Counting Rules.

2
https://www.socscistatistics.com/tests/ztest/

3 The Pareto distribution typically refers to an 80–20 rule–the distribution of 80% of an outcome among 20% of units. It is named for the Italian sociologist and economist, Vilfredo Pareto.
